# Wearable Cardioverter–Defibrillator-Measured Step Count for the Surveillance of Physical Fitness during Cardiac Rehabilitation

**DOI:** 10.3390/s21217054

**Published:** 2021-10-25

**Authors:** Boldizsar Kovacs, Flavia Müller, David Niederseer, Nazmi Krasniqi, Ardan M. Saguner, Firat Duru, Matthias Hermann

**Affiliations:** 1 Department of Cardiology, University Heart Center Zurich, 8091 Zurich, Switzerland; boldizsar.kovacs@usz.ch (B.K.); flavia.mueller@usz.ch (F.M.); ardan.saguner@usz.ch (A.M.S.); firat.duru@usz.ch (F.D.); matthias.hermann@usz.ch (M.H.); 2 GZO Regional Healthcare Center, 8620 Zurich, Switzerland; nazmi.krasniqi@gzo.ch; 3 Center for Integrative Human Physiology, University of Zurich, 8006 Zurich, Switzerland

**Keywords:** wearable cardioverter–defibrillator, cardiac rehabilitation, step count, accelerometer, heart failure

## Abstract

Background: The wearable cardioverter–defibrillator (WCD) has a built-in accelerometer, which allows tracking of patients’ physical activity by remote monitoring. It is unclear whether WCD-measured physical activity, step count, and heart rate correlate with established tools for the assessment of cardiopulmonary fitness such as the 6-min walk test (6MWT). Objective: To correlate measurements of patient physical activity through the WCD with a supervised 6MWT during in-patient cardiac rehabilitation (CR) and to allow their use as surrogate parameters of cardiopulmonary fitness in an out-patient setting. Methods: Consecutive patients with a history of WCD use treated at our center and an in-patient CR following an index hospitalization were included. Baseline characteristics, measurements of WCD accelerometer (median daily step count, median daily activity level), median daily heart rate, and clinically supervised 6MWT at admission and discharge of CR were obtained. Results: Forty-one patients with a mean age of 55.5 (±11.5) years were included. Thirty-five patients (85.4%) were male and 28 patients (68%) had a primary prophylactic WCD-indication. The most common underlying heart diseases were ischemic heart disease (24 patients 58.6%) and dilated cardiomyopathy (13 patients, 31.7%). Median CR duration was 20 (IQR 19.75–26.25) days. 6MWT distance increased from a mean of 329 m (±107) to 470 m (±116) during CR (*p* < 0.0001). The median daily step count and activity level increased significantly, from 5542 steps (IQR 3718–7055) to 8778 (IQR 6229–12,920, *p* < 0.0001) and median 117 *×* 10^6^ (IQR 96 *×* 10^6^–142 *×* 10^6^) threshold value exceedance (TVE) to 146 *×* 10^6^ TVE (IQR 110 × 10^6^–169 × 10^6^, *p* < 0.0001), respectively. The median heart rate was 74.9 bpm (IQR 65.8–84.5) and 70.2 (IQR 64.1–77.3, *p* = 0.09) at admission and discharge, respectively. Of all three parameters, median daily step count showed the best correlation to the results of the 6MWT at admission and discharge (r = 0.32, *p* = 0.04 and 0.37, *p* = 0.02, respectively). Conclusions: Remote monitoring of median daily step count as assessed by the WCD’s accelerometer showed positive correlation with the 6MWT and could serve as a surrogate for cardiopulmonary exercise capacity. Assessment of daily step count and activity level measured remotely by the WCD could help to tailor optimal exercise instruction for patients not attending CR.

## 1. Introduction

Cardiac rehabilitation (CR) is one of the pillars of the treatment of cardiovascular diseases to increase survival, decrease hospitalization and improve quality-of-life [1,2,3,4,5]. Although several large-scale trials have proven its benefit, it is still systematically underutilized [2,6,7,8].

A major component of comprehensive CR is physical activity to improve functional capacity and cardiopulmonary fitness. The 6 min walk test (6MWT) is an effective, reproducible yet simple measure of submaximal cardiopulmonary exercise capacity [9,10,11,12,13]. In addition, the 6MWT has been validated in the setting of CR [14].

The wearable cardioverter–defibrillator (WCD) offers protection from sudden arrhythmic death in patients at increased risk, but before implantable cardioverter defibrillator (ICD) implantation is warranted [15,16]. Patients enrolled in CR clinics after an acute cardiac event are therefore frequently candidates for its use. The current WCD model has been equipped with a built-in accelerometer able to perform a guided 6MWT, which has been shown the be accurate [17]. This function, however, has to be manually activated. The WCD can furthermore automatically measure daily physical activity and step count. These additional functions could naturally serve during outpatient follow-up of patients.

We therefore hypothesized that measurements of the WCD-accelerometer correlate with patients’ cardiopulmonary fitness assessed by 6MWT during treatment in structured CR clinics with the aim of expanding this function to outpatient follow-up.

## 2. Materials and Methods

Consecutive patients since the use of the WCD in Switzerland treated at the University Hospital of Zurich, in whom complete datasets were available, were screened for a history of WCD use and in-patient CR following index hospitalization. All patients for whom these inclusion criteria were present were included in this study. Electronic patient records were reviewed for baseline characteristics and follow-up data. WCD-specific data were provided by the manufacturer (ZOLL, Pittsburgh, Pennsylvania, USA). Informed consent was obtained from all patients. This study conforms to the Declaration of Helsinki as revised in 2013. The local ethics committee approved this study (Approval Number 2017-01044).

Patient data were pseudonymized and compiled in the REDcap Software (Vanderbilt, Nashville, TN, USA). The following baseline characteristics were gathered: age, body mass index (BMI, kg/m^2^), gender, comorbidities, left ventricular ejection fraction (EF), cardiovascular medication, underlying heart disease, and indication for WCD. Follow-up data included WCD interventions, follow-up EF, length of CR clinic stay, medication at discharge from CR clinical, and follow-up duration. The manufacturer provided patient-level WCD data from the LifeVest Network (mean daily wear-time, total daily wear-time, etc.) in a comprehensive pseudonymized database.

A detailed technical description of the WCD has been provided elsewhere [16]. In short, the WCD is a wearable defibrillator, which continuously monitors the heart rate with four electrocardiographic electrodes. Additionally, there are three defibrillation electrodes able to deliver a shock with up to 150 Joules, if indicated. A more recent feature of the WCD is the assessment of physical activity and heart rate. It has a built-in three-dimensional accelerometer with the capacity of measuring step count and patient activity, and measures and stores the heart rate in 5 min intervals [18].

To address our hypothesis, the following additional data were obtained from the manufacturer as assessed by the WCD: (1) Activity level; (2) Pedometer data (i.e., median step count); (3) median heart rate; each in 5 min intervals for the whole duration of CR clinical stay. For the first and last complete day (i.e., 24 h) in CR clinic, median values were calculated for all three parameters. Activity level was defined as any movement above the preset accelerometer threshold, i.e., any movement above this threshold increased the counter which then resulted in a numeric value, i.e., count of times the threshold was reached (threshold value exceedances in 10^6^ per 24 h, TVE). The step count was measured using the same accelerometer, and the heart rate was measured using the built-in electrodes of the WCD.

Correlation of these three variables to the 6MWT at admission and discharge from CR clinical was assessed. The 6MWT requires a 30 m long walking route (e.g., a hallway) and for 6 min the patient has to walk as quickly as possible on a flat, hard surface. The examiner measures the distance covered (in meters) and this serves as a reference value. As the test is performed at a submaximal self-paced level, it reflects the functional exercise level for daily physical activity of the patient [19].

A descriptive statistical analysis was performed on the available data set. Categorical variables are reported as percentage, continuous variables as means (±standard deviation) or medians (range), as appropriate. The Wilcoxon signed-rank test was performed to assess the differences in median daily step count, heart rate and activity level at the first and the last day of CR, and the 6MWT at CR admission and discharge due to skewed data. A two-sided *p*-value < 0.05 was considered statistically significant. Additionally, the correlation between the 6MWT and the three studied WCD parameters at admission and at discharge were calculated. All analyzes were performed using Python version 3.8.5 (Python Software Foundation, Land).

## 3. Results

Forty-one patients were included in this study (Table 1). The mean age was 56 years (±12), 35 patients (86%) were male, and 28 patients (68%) had a primary prophylactic WCD-indication. The most common underlying heart disease was ischemic heart disease in 24 patients (59%).

Results are reported as frequencies (percentage) or mean ± standard deviation or median (interquartile range); Abbreviations: COPD = chronic obstructive pulmonary disease; EF = ejection fraction; ACE Inhibitors = angiotensin converting enzyme inhibitors; VKA = vitamin K antagonist; NOAC = new oral anticoagulant or “non vitamin K antagonist” oral anticoagulant; AVRT = atrioventricular reentrant tachycardia; AVRNT = atrioventricular nodal reentrant tachycardia.

The main indication for WCD was low EF deemed at increased arrhythmogenic risk in 32 patients (78%), see also Table 2. Patients wore the WCD on average over 95 days with a high therapy adherence of 22.4 h per day mean daily wear-time (Table 2). No WCD treatments were administered in this cohort.

Total number included in the table n = 41; patients could have ≥1 indication for WCD use; Results are reported as frequencies (percentage) or mean ± standard deviation; Abbreviations: EF = ejection fraction; HTX = heart transplantation; MI = myocardial infarction; PCI = percutaneous coronary intervention; CABG = coronary artery bypass grafting; WCD = wearable cardioverter defibrillator.

### Change in Physical Activity during Cardiopulmonary Rehabilitation

**Figure 1:**
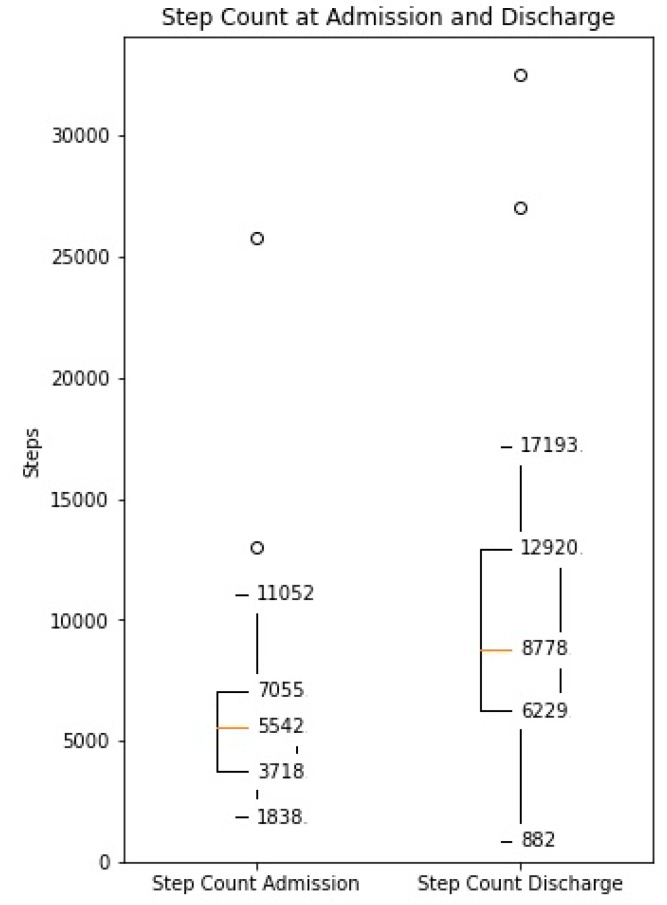
Median daily step count measured by the WCD at the time of admission and discharge to the CR clinic (Increase from median 5542 (IQR 3718–7055) to median 8778 (IQR 6229–12,920), *p* < 0.001).

**Figure 2:**
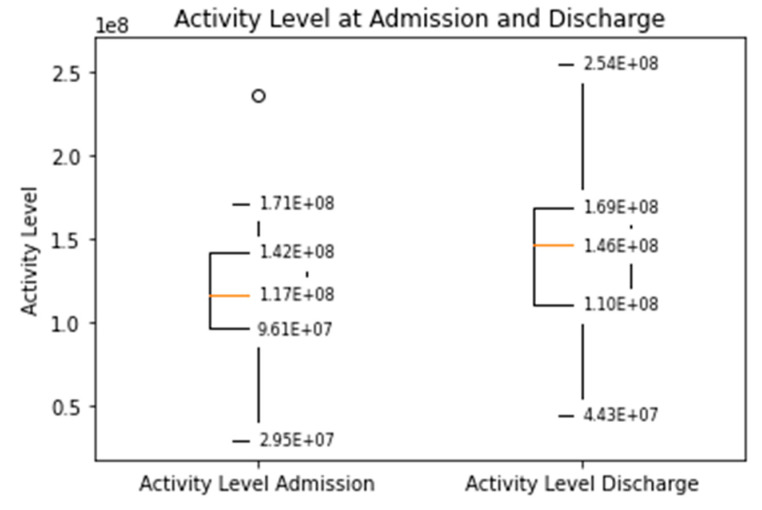
Median daily activity level measured by the WCD at the time of admission and discharge to the CR clinic (Increased from a median 117 × 10^6^ (IQR 96 × 10^6^–142 × 10^6^) TVE to 146 × 10^6^ (110 × 10^6^–169 × 10^6^), *p* < 0.001).

**Figure 3:**
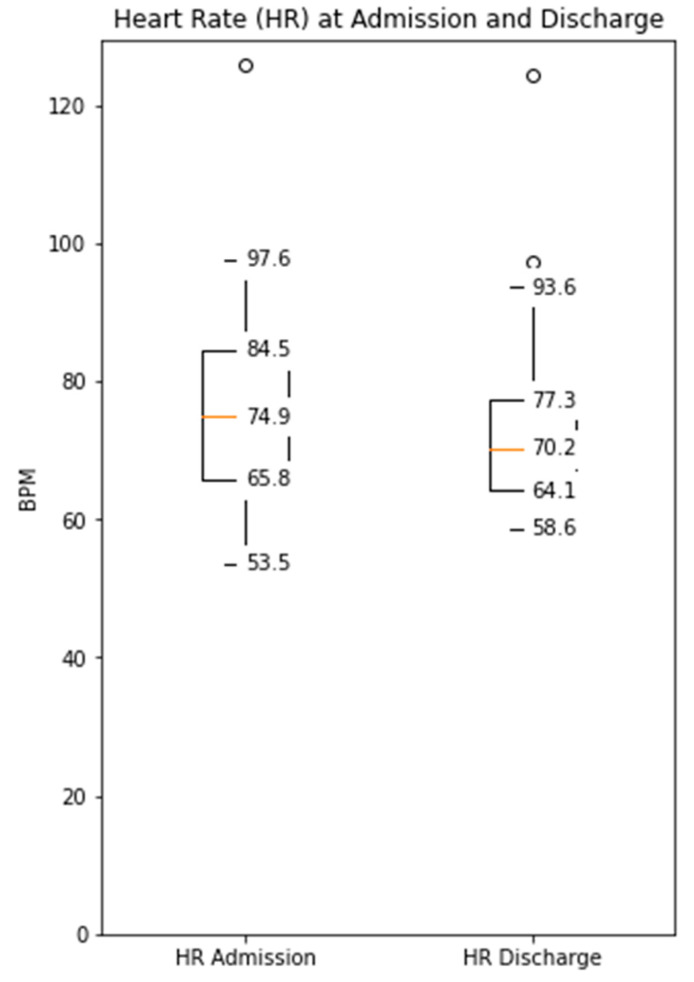
Median daily heart rate measured by the WCD at the time of admission and discharge to the CR clinic (Decreased from a median 74.9 bpm (IQR 65.8–84.5) to 70.2 (IQR 64.1–77.3), *p* = 0.09).

The distance covered during the 6MWT at the time of CR clinic admission significantly increased from a mean of 329 m (±107) to 470 m (±116) at the time of discharge (*p* < 0.0001, Supplementary Figure S1). The median daily step count as assessed by the WCD’s accelerometer significantly increased from 5542 (IQR 3718–7055) on the day of admission to a median of 8778 (IQR 6229–12,920, *p* < 0.0001) on the day of discharge (Figure 1). Similarly, the activity level significantly increased from a median 117 × 10^6^ (IQR 96 × 10^6^–142 × 10^6^) TVE on the day of admission to 146 × 10^6^ (110 × 10^6^–169 × 10^6^), *p* < 0.0001) TVE on the day of discharge (Figure 2). The median heart rate numerically decreased from 74.9 bpm (IQR 65.8–84.5) on the day of admission to 70.2 (IQR 64.1–77.3, *p* = 0.09) on the days of discharge (Figure 3).

For each WCD dataset, correlation to the validated 6MWT at the time of admission and discharge from CR clinic was calculated.

**Figure 4:**
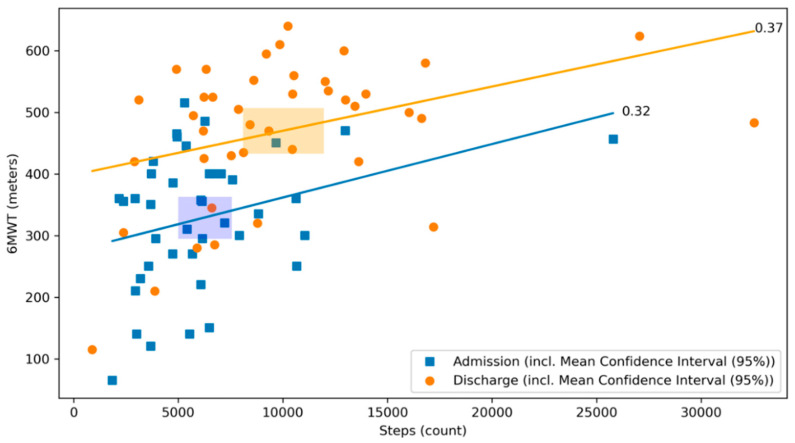
Scatterplot of 6MWT and step count at admission and discharge with respective correlation coefficients. The relationship between the 6MWT and the step count at admission (blue) and discharge (orange) of the CR stay are illustrated. The graph visualizes their respective correlation coefficients. Blue dots represent patients at admission, whereas orange dots represent patients at discharge. The lines depict the correlation coefficient between the 6MWT and the step count at admission in blue and at discharge in orange. The rectangles represent the mean confidence intervals of the respective means (r = 0.32, *p* = 0.04 and 0.37, *p* = 0.02, respectively).

The best correlation with the 6MWT was found for the step count, both at admission and discharge with significant and comparable correlation coefficients (r = 0.32, *p* = 0.04 and 0.37, *p* = 0.02, respectively (Figure 4), meaning that the daily median step count increased to a similar extent from the day of admission to the day of discharge as the distance covered by the 6MWT at the same time points.

**Figure 5:**
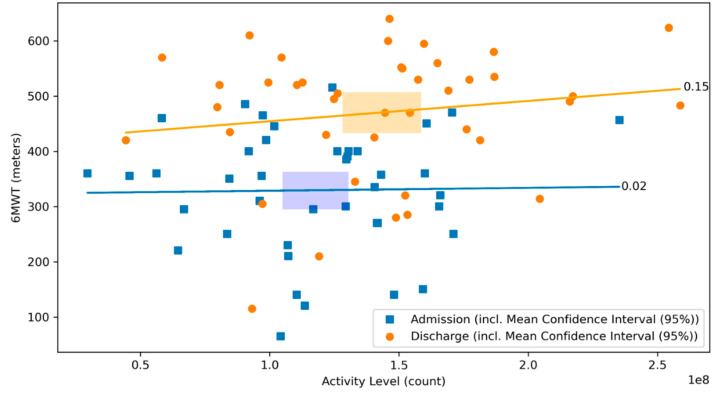
Scatterplot of 6MWT and activity level at admission and discharge with respective correlation coefficients. The relationship between the 6MWT and the activity level at admission (blue) and discharge (orange) of the CR stay are illustrated. The graph visualizes their respective correlation coefficients. Blue dots represent patients at admission, whereas orange dots represent patients at discharge. The lines depict the correlation coefficient between the 6MWT and the activity level at admission in blue and at discharge in orange. The rectangles represent the mean confidence intervals of the respective means (r = 0.02, *p* = 0.90 and r = 0.15, *p* = 0.35, respectively).

**Figure 6:**
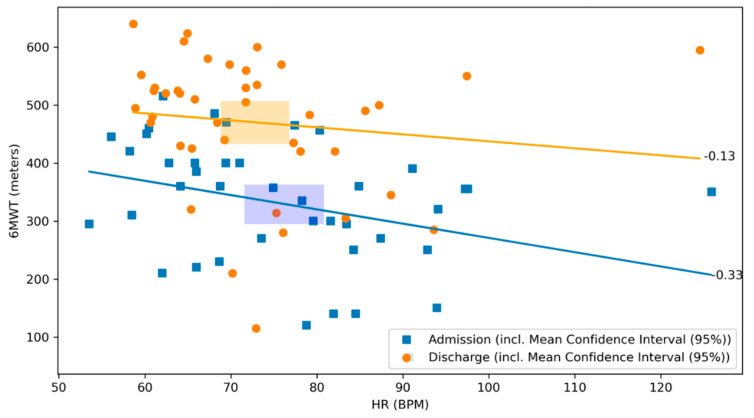
Scatterplot of 6MWT and heart rate at admission and discharge with respective correlation coefficients. The relationship between the 6MWT and the heart rate at admission (blue) and discharge (orange) of the CR stay are illustrated. The graph visualizes their respective correlation coefficients. Blue dots represent patients at admission, whereas orange dots represent patients at discharge. The lines depict the correlation coefficient between the 6MWT and the heart rate at admission in blue and at discharge in orange. The rectangles represent the mean confidence intervals of the respective means (r = −0.33, *p* = 0.03 and r = −0.13, *p* = 0.41, respectively).

There was no significant correlation between the 6MWT and the daily activity level neither at admission, nor at discharge (r = 0.02, *p* = 0.90 and r = 0.15, *p* = 0.35, respectively; Figure 5). There was a negative correlation between the distance covered by 6MWT and the heart rate on admission (r = −0.33, *p* = 0.03), whereas no significant correlation was observed at discharge (r = −0.13, *p* = 0.41, respectively; Figure 6).

## 4. Discussion

For the first time, this study evaluated the automatically collected data set measured by the WCD during CR to assess improvements of cardiopulmonary fitness. We demonstrate that patients’ step count shows a good correlation to the distance covered by the 6MWT and may be used as a surrogate for cardiopulmonary fitness.

Patients prescribed with a WCD frequently undergo in-patient CR after their index hospitalization in Switzerland. In addition to the protection it offers from sudden arrhythmic death, a valuable function is its built-in accelerometer to assess physical fitness after an acute cardiac event. In this study, for the first time, we demonstrated that all automatic measurements of the WCD-median daily step count, daily activity level, and median heart rate each showed individually different dynamics over the course of CR. We additionally assessed the development of cardiopulmonary fitness during CR separately by 6MWT in correlation to each individual WCD parameter to be able to quantify the possibility of each parameter functioning as a stand-alone surrogate for cardiopulmonary fitness.

Our aim was to assess whether the daily measurements of the WCD’s accelerometer could serve as a surrogate for the increase in cardiopulmonary fitness during CR. Indeed, we found a significant increase in step count and activity level over the course of CR. The median step count showed a good correlation to the 6MWT performed in the CR. These findings suggest that, in addition to the WCD-directed 6MWT, the simple daily measurement of step count could be used as a surrogate for patients’ physical fitness. Of note, the WCD has an established function which allows to perform a guided 6MWT, which has already shown good correlation with in-clinic results [17].

The continuous and reliable assessment of physical fitness could be of significant clinical benefit, as recent studies suggested that more physically fit patients have a lower risk of sudden cardiac events during the use of either an ICD [20] or WCD [10]. In the first week after the WCD prescription, decreased physical activity was associated with a higher risk to experience an adequate shock. Indeed, patients with a mean daily step count below 3637 had a highly increased odds ratio for adequate shock (OR = 4.29, 95% CI = 2.58–7.15) in a large cohort consisting of 4057 patients with mixed indications for WCD use [10].

Another study examined a cohort of early post-myocardial infarction patients with severely reduced EF ≤ 35% [21]. Trip et al. assessed the evolution of mean daily step count of 1952 patients over the course of 90 days of WCD use. They found an increase in median daily step count from ~3800 to ~6100 during their study period. The authors, however, did not assess what percentage of patient participated in CR. In comparison, our study population, consisting of patients with mixed indication for WCD and a mean EF of 26% with in-patient CR, had an increase of the median daily step count from 5542 to 8778 over the course of median 20 days.

Burch et al. measured the change of distance covered by the 6MWT, as assessed in the clinic and by the WCD [17]. His group predominantly examined a heart failure population with an average EF of 23% over the course of eight weeks of WCD use. During the study period, the mean distance covered by the 6MWT was 554 steps without significant increase [17]. In our cohort, there was a significant increase in the distance covered by the 6MWT (329 m to 470 m, respectively) with good correlation to the median daily step count. While the difference in units measured does not allow a direct comparison between the studies, the significant increase in the outcome of the 6MWT and median daily step count not only support the reliability and validity of the accelerometer function of the WCD, but in addition highlight the benefit of structured CR.

The activity level as assessed by the WCD also increased significantly during the study period, albeit not in a similar manner as the 6MWT. This can have several reasons, the most likely being that a low threshold lead to a high count in the activity level despite only little movement or change in body position, and while low-intensity activities compared to no activity may have a beneficial effect of cardiovascular outcomes [22,23], moderate intensity training offers better improvements in health status [24]. Furthermore, the sensitivity level programmed may significantly influence the counted activity level which impedes interpretation of increasing or decreasing values.

As a lower resting heart rate is associated with better cardiovascular outcomes [25], we hypothesized that increasing physical fitness (i.e., as assessed by the 6MWT) would lead to a decreased heart rate. While, at admission, we found a significant inverse correlation between the distance covered and the median daily heart rate, this could not be replicated. We measured median heart rates during whole days and not simply resting heart rate which is subject to several confounders which may explain these results. Moreover, up-titration of heart failure medication (i.e., betablockers and ivabradine) is indicated as soon as achievable after index cardiac hospitalization likely also affecting our analyses. Patients who do not tolerate higher beta blocker dosages during CR may represent a sicker patient population who are likely to have more impaired physical fitness.

Our findings suggest that automatic measurement of daily step count by the WCD could replace the 6MWT in everyday clinical practice. Patients not entering remote monitoring of patients with a WCD could allow to track the evolution of their cardiopulmonary fitness and intervene (i.e., offer out- or in-patient CR) if signs of a decline arise, especially in case of patients who do not participate in in-patient CR. Remote monitoring could be implemented similarly to the use of remote monitoring of thoracic impedance to predict heart failure decompensations, which has increasing implications in clinical practice [26].

The main limitation of this study was the retrospective nature and small population, introducing the possibility of bias. However, the correlated variables were robust and allow for precise analyses. Unknown confounders cannot be excluded however. No statement can be made regarding the role of the WCD’s accelerometer according to subgroups (i.e., ICM) owing to the low number of patients in each subgroup. Moreover, only measurements at the time of admission and discharge were compared. However, these reliably depict improvement in fitness during CR which is the most important outcome measure.

## 5. Conclusions

Remote monitoring of median daily step count as assessed by the WCD’s accelerator showed positive correlation with the 6MWT and could serve as a surrogate for cardiopulmonary exercise capacity. This may offer clinicians the possibility of intervening if a decline or lack of improvement in daily step count and activity level measured remotely by the WCD is observed after an index hospitalization in patients not admitted for CR.

## Figures and Tables

**Table 1:** Baseline characteristics of study population.

Baseline Characteristics	n (%) or Mean ± SD or Median (IQR)
Number	41 (100)
Female	6 (14.6)
Age (years)	55.5 (11.5)
BMI (kg/m^2^)	26.7 (5)
EF before WCD use	26.3 (8.6)
Primary prophylactic WCD use	28 (68)
Secondary prophylactic WCD use	13 (32)
Index hospitalization duration (days)	15 (12–20.25)
Rehabilitation clinic hospitalization duration (days)	20 (19.75–26.25)
**Underlying heart diseases**	
Ischemic heart disease	24 (58.6)
Dilated cardiomyopathy	13 (31.7)
Valvular heart disease	6 (14.6)
Hypertensive heart disease	3 (7.3)
Myocarditis	2 (2.4)
Other	2 (2.4)
**Comorbidities**	
Hypertension	18 (43)
Dyslipidemia	11 (26.8)
Diabetes mellitus 1 or 2	8 (19.5)
COPD	4 (9.8)
CKD (KDIGO 3b or higher)	3 (7.3)
Current smoker	14 (34.2)
**Cardiovascular therapy**	
ACE inhibitors or angiotensin II receptor blocker	39 (95.1)
Aldosterone antagonists	24 (58.6)
Beta blockers	38 (92.7)
Diuretics	30 (73.2)
Antiplatelet agents	28 (68.3)
Statins	27 (65.9)
VKA	13 (31.7)
NOAC	4 (9.8)
Amiodarone	6 (14.6)
Calcium channel blockers	2 (4.9)
**Preexisting supraventricular tachyarrhythmias**	
Atrial fibrillation	9 (22)
Atrial flutter	5 (12.2)
AVNRT	2 (4.9)
Other	2 (4.9)
None	25 (61)

**Table 2:** Indication for WCD use and WCD data.

Indication for WCD	n (%)	n ± SD
Low EF and previous ventricular arrhythmia or high arrhythmogenic risk	32 (78)	
After MI	10 (24.4)	
After CABG	10 (24.4)	
After PCI	8 (19.5)	
After percutaneous valve repair	2 (4.9)	
Bridge to HTX	1 (2.4)	
**WCD wear-time**		
Mean daily wear-time (hours)		22.4 (2.8)
Mean total wear-time (days)		95.9 (56.1)

## Data Availability

Data used in this study can be made available upon reasonable request.

## Supplementary Materials

The following are available online at https://www.mdpi.com/article/10.3390/s21217054/s1, Figure S1, 6MWT at Admission and Discharge. Admission and discharge levels are from the cardiopulmonary rehabilitation clinic stays.
