# Supplementary material for: Wearable Cardioverter–Defibrillator-Measured Step Count for the Surveillance of Physical Fitness during Cardiac Rehabilitation
Source: Sensors (Basel). 2021 Oct 25;21(21):7054. doi: 10.3390/s21217054 (PMC8588232; doi:10.3390/s21217054)
Supplement: Supplementary file 1 [file sensors-21-07054-s001.zip › Supplementary Figure S1.pdf]

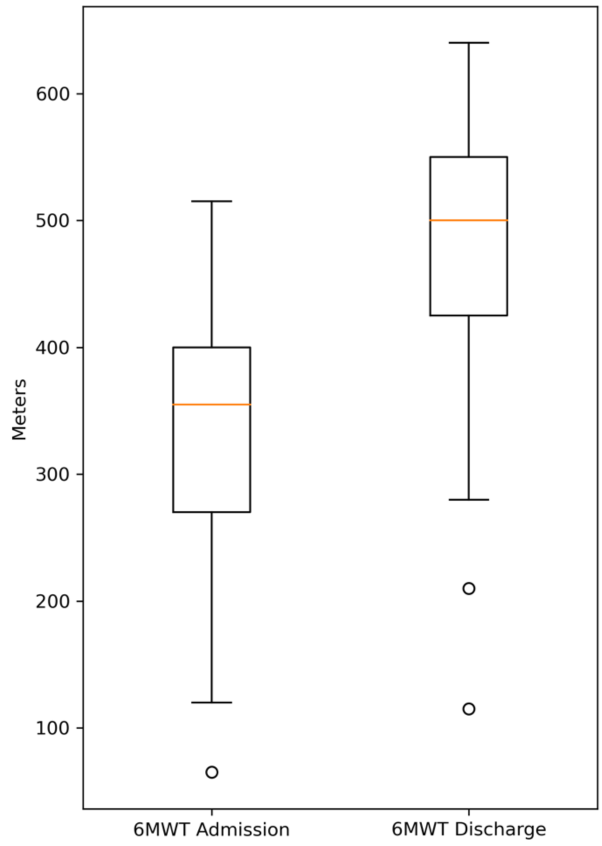

Figure S1: 6MWT at Admission and Discharge. Admission and discharge levels are from the cardiopulmonary rehabilitation clinic stays.
